# Development of an Enzyme-Linked Immunosorbent Assay to Determine the Expression Dynamics of Ebola Virus Soluble Glycoprotein during Infection

**DOI:** 10.3390/microorganisms8101535

**Published:** 2020-10-06

**Authors:** Wakako Furuyama, Andrea Marzi

**Affiliations:** Laboratory of Virology, Division of Intramural Research, National Institute of Allergy and Infectious Diseases, National Institutes of Health, Hamilton, MT 59840, USA; wakako.asada@nih.gov

**Keywords:** filovirus, EBOV, sGP, ELISA, animal models

## Abstract

Ebola virus (EBOV) is a highly pathogenic virus with human case fatality rates of up to 90%. EBOV uses transcriptional editing to express three different glycoproteins (GPs) from its GP gene: soluble GP (sGP), GP, and small sGP (ssGP). The molecular ratio of unedited to edited mRNA is about 70% (sGP): 25% (GP): 5% (ssGP), indicating that sGP is produced more abundantly than GP. While the presence of sGP has been confirmed in the blood during human EBOV infection, there is no report about its expression dynamics. In this study, we developed an EBOV-sGP-specific sandwich enzyme-linked immunosorbent assay (ELISA) using two different available antibodies and tested several animal serum samples to determine the concentration of sGP. EBOV-sGP was detected in nonhuman primate serum samples as early as 4 days after EBOV infection, correlating with RT-qPCR positivity. This ELISA might be further developed into a diagnostic tool for detection of EBOV in patients. Furthermore, this study provides insights into the expression dynamics of sGP during infection, which are important to decipher the function that sGP plays during infection.

## 1. Introduction

Ebolaviruses, members of the family *Filoviridae*, are filamentous, enveloped, nonsegmented, negative-stranded RNA viruses with a genome encoding seven genes. Six distinct species are known in the genus *Ebolavirus*: *Zaire ebolavirus, Sudan ebolavirus, Taï Forest ebolavirus, Bundibugyo ebolavirus, Bombali ebolavirus,* and *Reston ebolavirus*, represented by Ebola virus (EBOV), Sudan virus (SUDV), Taï Forest virus (TAFV), Bundibugyo virus (BDBV), Bombali virus (BOMV), and Reston virus (RESTV), respectively [1,2]. The 2013–2016 EBOV epidemic in West Africa, which originated in Guinea and spread to other countries, resulted in more than 10,000 fatalities among at least 28,000 cases [3]. Today, there is an ongoing EBOV outbreak in the Democratic Republic of the Congo (DRC) [4], a country dealing with EBOV outbreaks since 2018 [5,6]. The epidemic and the DRC outbreaks accelerated efforts to develop antiviral strategies and vaccines. However, they also highlighted the urgent need for development of diagnostic assays.

Ebolaviruses express a single transmembrane glycoprotein (GP), which includes the receptor binding (GP1) and viral fusion (GP2) subunits [7,8]. However, the GP gene encodes for three different proteins, expression of which is controlled by transcriptional editing at the site of seven adenosine residues (7A). When the viral polymerase encounters this editing site during transcription, in 70−80% of cases a primary unedited 7A transcript is produced, which leads to production of soluble GP (sGP). The remaining 20–30% are left for the edited 8A full-length GP which is expressed on the virion surface, and the edited 9A small sGP (ssGP) [9,10]. Even though sGP is transcribed and expressed at a higher level compared to GP and is detected in the blood of EBOV-infected animals and humans, only few diagnostic tests targeting sGP exist [11,12,13].

In this study, we developed an EBOV-sGP-specific sandwich ELISA for detection of sGP in EBOV-infected cell culture supernatants and animal serum samples using two EBOV-specific antibodies. Furthermore, establishment of a standard curve for this sGP sandwich ELISA resulted in determination of the sGP concentration, which revealed the sGP expression dynamics in the serum of EBOV-infected nonhuman primates (NHPs) over time.

## 2. Materials and Methods

### 2.1. Ethics Statement

All infectious work was performed at the required containment level at the Integrated Research Facility, Rocky Mountain Laboratories (RML), Division of Intramural Research (DIR), National Institute of Allergy and Infectious Disease (NIAID), National Institutes of Health (NIH). The animal work was approved by the Institutional Animal Care and Use Committee (IACUC) and performed according to the guidelines of the Association for Assessment and Accreditation of Laboratory Animal Care, International and the Office of Laboratory Animal Welfare. All procedures on animals were carried out by trained and certified personnel following standard operating procedures (SOPs) approved by the Institutional Biosafety Committee (IBC). Humane endpoint criteria in compliance with IACUC-approved scoring parameters were used to determine when animals should be humanely euthanized.

### 2.2. Cells and Viruses

African green monkey kidney (Vero E6), human hepatoma (Huh7), and human embryonic kidney 293 (HEK293) cells were grown in Dulbecco’s modified Eagle’s medium (DMEM) (Sigma-Aldrich) containing 10% fetal bovine serum (FBS), 2 mM L-glutamine, 50 U/mL penicillin, and 50 μg/mL streptomycin (all from Thermo Fisher Scientific, USA). The FreeStyle 293-F (293F) (Invitrogen) cells were cultured in FreeStyle 293 Expression medium (Thermo Fisher Scientific) according to the manufacturer’s instructions. EBOV expressing GFP (EBOV-GFP) [14], EBOV-Makona Guinea C05 (EBOV-C05) [15], EBOV-Makona Guinea C07 (EBOV-C07) [15], mouse-adapted EBOV (MA-EBOV) [16], and guinea pig-adapted EBOV (GPA-EBOV) [16] were propagated in Vero E6 cells and stored at −80 °C. Recombinant EBOV with a mutated editing site in GP from 7A (AAAAAAA) to sGP-knockout (AAGAAGAA) (EBOV-sGP-KO) was recovered from the full-length EBOV plasmid containing the mutated GP gene and propagated as previously described [17]. Briefly, HEK293 cells 60% confluent in 6-well plates were transfected with EBOV helper plasmids (125 ng pCAGGS-NP, 125 ng pCAGGS-VP35, 75 ng pCAGGS-VP30, 1 ug pCAGGS-L, and 250 ng pCAGGS-T7) and 250 ng EBOV-sGP-KO plasmid using Transit-LT1 (Mirus). At 24 h post-transfection, the medium was replaced, and after 7 days the supernatant was transferred onto Vero E6 cells. Upon development of cytopathic effect, the supernatant was collected and stored at −80 °C.

### 2.3. Generation of Recombinant sGP

Expression plasmids for pCAGGS-EBOV (Mayinga), SUDV (Boniface), BDBV (Butalya), or RESTV (Pennsylvania 89) sGP were transfected in 293F cells according to the manufacturer’s instructions. After incubation for 4 days, the supernatant was cleared from cell debris by centrifugation twice at 3500 rpm for 10 min at 4 °C. The supernatants containing each sGP were collected and concentrated in Amicon Ultra 30 K filters (Millipore) to a final concentration of 1–2 mg/mL and stored at −80 °C. Supernatant of nontransfected 293F cells was also concentrated using the same method and used as a control protein. Expression of each sGP was confirmed by mixing the concentrated protein 1:1 with sodium dodecyl sulfate-polyacrylamide gel electrophoresis (SDS-PAGE) sample buffer containing 20% β-mercaptoethanol and incubated at 99 °C for 10 min. SDS-PAGE with all samples was performed in parallel on Tris-Glycine eXtended (TGX) criterion pre-cast gels (Bio-Rad Laboratories). The gel was processed for silver staining, using the Pierce Silver Stain Kit (Thermo Fisher Scientific, USA).

### 2.4. Generation of Ebola virus-like Particles (Ebola VLPs)

For generation of Ebola VLPs, HEK293T cells were transfected with equal amounts of the expression plasmids encoding EBOV-GP, matrix protein (VP40), and nucleoprotein (NP) using TransIT LT-1 (Mirus) according to the manufacturer’s instructions. Forty-eight hours (h) after transfection, the culture supernatant was harvested and centrifuged twice at 3500 rpm for 10 min at 4 °C to remove cell debris. VLPs were precipitated through a 20% sucrose cushion by centrifugation at 28,000 rpm for 2 h at 4 °C with an SW32Ti rotor (Beckman). Pelleted VLPs were resuspended in phosphate-buffered saline (PBS) and stored at −80 °C.

### 2.5. Growth Kinetics

Vero E6, HEK293, or Huh7 cells were grown to confluency in a 12-well plate and infected in triplicate with EBOV-GFP, EBOV-sGP-KO, EBOV-C05, and EBOV-C07 (multiplicity of infection (MOI) 0.01). After 1 h incubation at 37 °C, the cells were washed 3 times with plain DMEM, and DMEM containing 2% FBS was added. Supernatant samples were collected at 0, 24, 48, 72 and 96 h post-infection and stored at −80 °C. The titer of supernatant samples was determined by a 50% tissue culture infectious dose (TCID_50_) assay on Vero E6 cells.

### 2.6. Western Blot Analysis

Samples were generated from each EBOV stock produced in Vero E6 cells by mixing a sample 1:1 with SDS gel electrophoresis sample buffer containing 20% β-mercaptoethanol and heated to 99 °C for 10 min. SDS-PAGE and transferred Trans-Blot polyvinylidene difluoride membrane (Bio-Rad Laboratories) with all samples was performed as described elsewhere [18]. The membrane was blocked for 3 h at room temperature in PBS with 3% powdered milk and 0.05% Tween 20 (Thermo Fisher Scientific). Protein detection was performed using anti-EBOV GP (ZGP42/3.7 or ZGP 12/1.1, 1 μg/mL) [19] and horse-raddish peroxidase (HRP)-labeled secondary antibody staining using anti-mouse Immunoglobulin G (IgG; 1:10,000) (Jackson ImmunoResearch). The blots were imaged using the SuperSignal West Pico chemiluminescent substrate (Thermo Fisher Scientific) and a FluorChem E system (ProteinSimple).

### 2.7. Serum Samples and Virus Load

Five-week-old female CD1 mice and Hartley strain guinea pigs were challenged with 10 plaque-forming unit (PFU) of MA- or GPA-EBOV, and the serum samples were collected 5 days before (day −5) or after (day 5) inoculation. Serum samples from 14 EBOV-infected rhesus macaques (*Macaca mulatta*) were used [15]. Virus load data from whole blood RNA samples from these 14 NHPs were generated following previously established protocols [20]. Serum samples from all EBOV-infected animals were gamma-irradiated for inactivation following SOPs approved by the IBC and used in the BSL2.

### 2.8. Establishment of the sGP Sandwich ELISA

NUNC Maxisorp Immunoplates were coated with 50 μL of 1 μg/mL ZGP42/3.7 at 4 °C overnight and then washed once with PBS containing 0.05% Tween 20 (PBST). The plate was blocked with 3% skim milk (190 μL/well) for 3 h at room temperature. After washing once with PBST, 50 μL of appropriately diluted serum or plasma samples or the concentrated sGPs in PBS containing 1% skim milk were added and incubated overnight at 4  °C. After washing 3 times with PBST, 50 μL of 1 μg/mL Rabbit anti-EBOV sGP pAb (IBT BIOSERVICES; 0365-001) was added and incubated for 1 h at room temperature. After washing 3 times with PBST, the bound antibodies were labeled by using 50 μL of 1:1000 peroxidase AffiniPure Donkey Anti-Rabbit IgG (H + L) (Jackson ImmunoResearch) diluted in 1% skim milk in PBST. After incubation for 1 h at room temperature and 3 PBST washes, 50 μL of KPL ABTS Peroxidase substrate solution mix (SeraCare Life Sciences) was added to each well, and the mixture was incubated for 15 min at room temperature. The optical density (OD) at 405 nm was measured. The standard curve for the sGP sandwich ELISA was carried out using serial dilutions of recombinant EBOV-sGP.

## 3. Results

### 3.1. Establishment and Specificity of the sGP Sandwich ELISA

**Figure 1:**
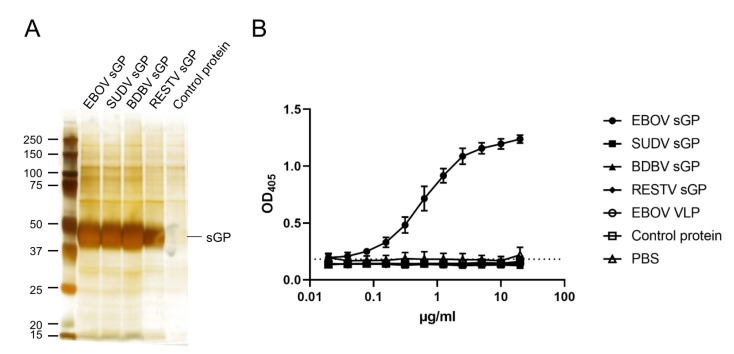
Establishment of the Ebola virus (EBOV) soluble glycoprotein (sGP) sandwich ELISA. (**A**) Concentrated sGPs of EBOV, Sudan virus (SUDV), Bundibugyo virus (BDBV), and Reston virus (RESTV) were subjected to electrophoresis on an SDS-PAGE gel and stained with a silver staining kit. Supernatant of nontransfected 293F was concentrated and used as a control protein. (**B**) Sensitivity and specificity of the sandwich ELISA for quantifying EBOV-sGP were determined. Serial two-fold dilutions of each sGP were analyzed in triplicates. Average and standard deviation are shown.

In order to determine the amount of sGP present during infection in serum samples during EBOV infection, we set out to establish a sGP-specific sandwich ELISA. To this end, 293F cells were transfected with sGP expression plasmid, and expression and secretion of each sGP in the supernatant of the 293F cells were confirmed by silver staining (Figure 1A). Next, we established and optimized the conditions for the sGP-capture ELISA. To demonstrate that the sGP-capture ELISA specifically detects EBOV sGP, we performed the ELISA using concentrated sGPs of SUDV, BDBV, and RESTV. We also used Ebola VLPs consisting of GP, NP, and VP40 which were confirmed to express only full-length GP (data not shown). At sGP concentrations ranging from 0.02 to 20 μg/mL, the sandwich ELISA was able to detect only EBOV sGP but not sGPs of other ebolaviruses and Ebola VLPs (Figure 1B). The detection limit for EBOV sGP using this assay was approximately 0.07 to 0.1 μg/mL.

### 3.2. Analysis of sGP in EBOV-Infected Cell Supernatant

**Figure 2:**
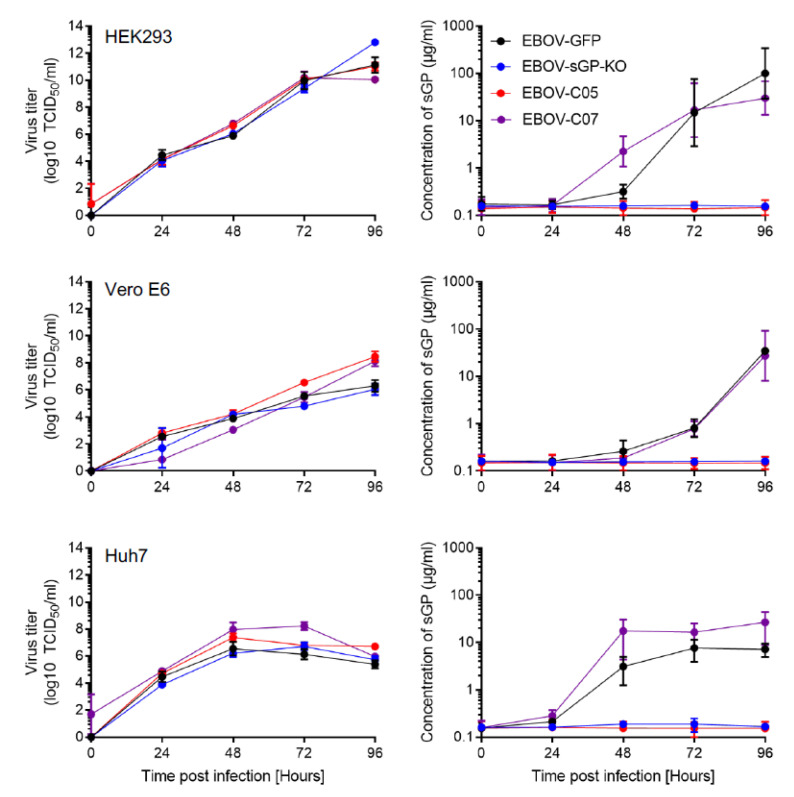
Determination of sGP concentrations in EBOV-infected cell culture supernatants. In vitro growth kinetics of each EBOV were performed with human embryonic kidney 293 (HEK293) (top), African green monkey kidney (Vero E6) (middle), or human hepatoma (Huh7) (bottom) cells. Each supernatant was used to determine the infectious EBOV titer (left panels) and amount of sGP (right panels). The geometric mean and standard deviation of one experiment performed in triplicates are shown.

**Figure 3:**
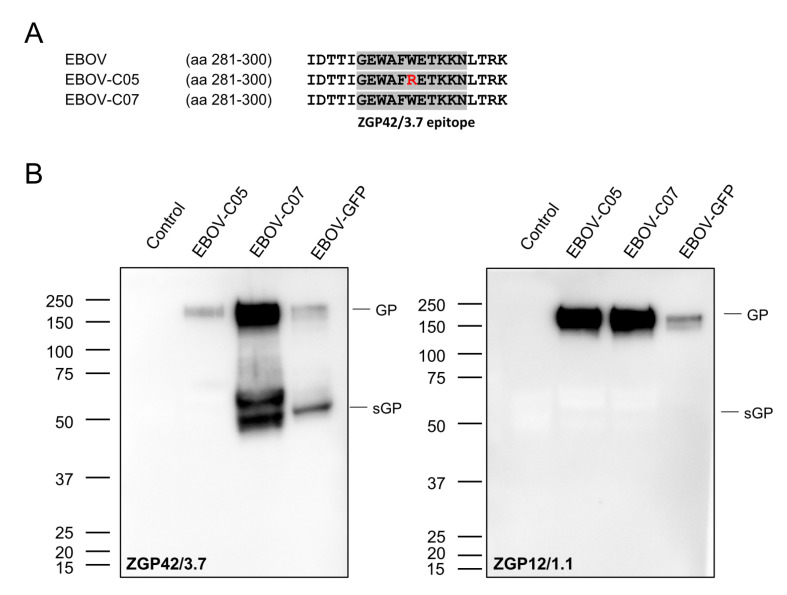
Binding ability of capture antibody ZGP42/3.7. (**A**) Comparison of protein sequences at the capture antibody binding site in GP amino acid (aa) 281–300 of EBOV variants. (**B**) Western blot analysis using the capture antibody ZGP42/3.7 (left panel) or the EBOV full-length GP-specific antibody ZGP12/1.1 (right panel).

In order to determine the kinetics of EBOV sGP expression in cell culture supernatants after infection, we performed growth kinetics with four different EBOVs: EBOV-GFP, EBOV-sGP-KO, EBOV-C05, and EBOV-C07. Vero E6, HEK293, and Huh7 cells were infected in triplicate with each virus at a MOI of 0.01, and samples were collected from the supernatant at 0, 24, 48, 72, and 96 h. The sandwich ELISA detected the sGP of EBOV-GFP and EBOV-C07 in all infected cell lines, and the dynamics were similar to the dynamics of the infectious virus titer (Figure 2). As expected, we could not detect sGP in the supernatant of EBOV-sGP-KO-infected cells, since this virus was mutated to not produce sGP. Surprisingly, no sGP was detected in the supernatant of cells infected with EBOV-C05 (Figure 2). In order to analyze if EBOV-C05 does not express sGP or if the antibody does not bind, viral RNA was extracted from the supernatant samples and the nucleotide sequences of the GP genes were determined. We confirmed that the sequence of the editing site matches the previously published sequences (EBOV AF086833.2; EBOV-C05 MG572232.1; and EBOV-C07 MG572233.1). We found that EBOV-C05 has a different amino acid (aa), Arg (R), at position 291 within the GP gene (Figure 3A), which is located in the binding epitope of capture antibody ZGP42/3.7 [21]. Western blot analysis confirmed that ZGP42/3.7 can be used for detection of GP and sGP of EBOV-C07 and EBOV-GFP, but this antibody does not bind to EBOV-C05 sGP and only very weakly to its GP (Figure 3B). As a control, the anti-EBOV GP-specific antibody ZGP12/1.1 was used, as it is known to bind only to EBOV GP. Indeed, GP was detected in all samples with the weakest signal in EBOV-GFP confirming that binding to ZGP42/3.7 is the problem, not GP or sGP expression.

### 3.3. Detection of EBOV-sGP in Infected Animals

**Figure 4:**
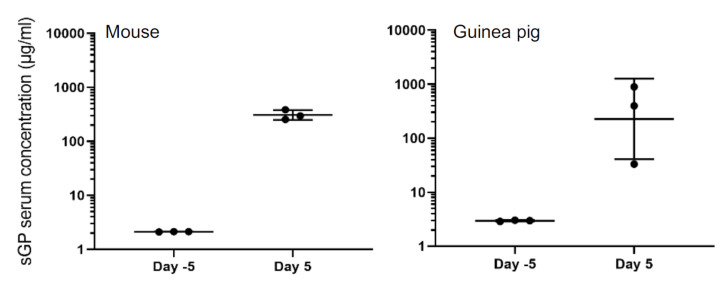
Detection of sGP in rodent serum samples. Measurement of sGP levels in the serum of EBOV-infected mouse and guinea pig samples. Groups of three mice or guinea pigs were inoculated with a lethal dose of mouse- or guinea pig-adapted EBOV, respectively. Serum samples were collected 5 days before (day −5) and after infection (day 5). Geometric mean and standard deviation are shown.

To investigate whether the sandwich ELISA could be used to detect the EBOV sGP in serum of infected animals, we first analyzed mouse and guinea pig serum samples collected 5 days prior to EBOV infection (day −5) and on day 5 after which is at a moderate-to-late stage of disease in these rodent models. We could not detect any EBOV sGP prior to challenge, but high amounts of EBOV sGP were detected on day 5 after virus infection in both rodent models (Figure 4), demonstrating that this ELISA can be used for serum samples.

**Figure 5:**
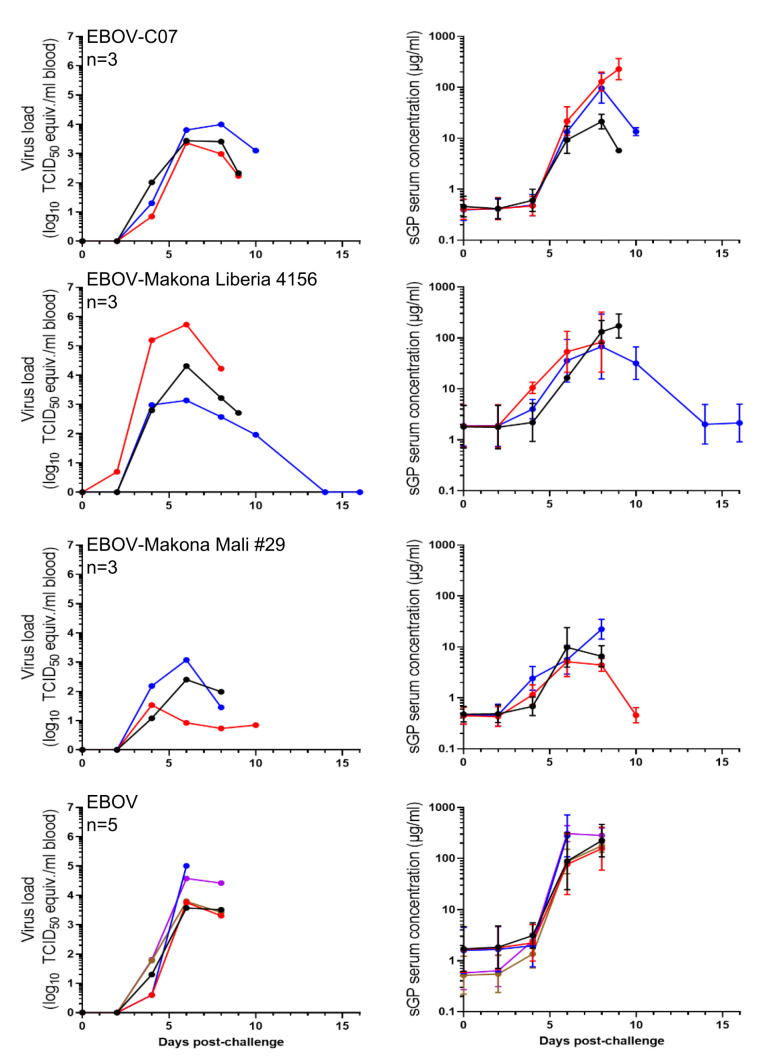
Detection of sGP in nonhuman primates (NHP) serum samples. Groups of NHPs were inoculated with each EBOV variant, and blood and serum samples were collected at every exam day and at the time of euthanasia. RT-qPCR (left panels) and ELISA (right panels) are shown. ELISA was performed in triplicate; geometric mean and standard deviation are shown.

Next, we wanted to determine the kinetics of sGP in serially obtained serum samples. We used samples collected from NHPs infected with EBOV, EBOV-C07, EBOV-Makona Mali #29, or EBOV-Makona Liberia 4156. EBOV-sGP was detected starting at day 4 post-infection (Figure 5 right panels), and the dynamics were similar to the virus load (Figure 5 left panels) as well as the infectious virus titer [15]. For most of the samples tested, sGP was detected in high concentrations (Figure 5). There was also no difference in OD measurements comparing heat-inactivated serum samples to nonheat-incativated samples (data not shown). Surprisingly, the system could detect sGP in samples with corresponding virus load as low as 100 TCID_50_ equivalents/mL of an EBOV-Mali #29-infected NHP (Figure 5, red line). Interestingly, this animal had its highest amounts of sGP in the blood on day 6, when there was no viremia [15], and only a low level of viral RNA in the blood was detected (Figure 5). This observation indicates that the sandwich ELISA is more sensitive than the previously published TCID_50_ assay [15] and has a similar sensitivity compared to the RT-qPCR. These results suggest that the assay shows high sensitivity and can be used for detection of EBOV sGP from multiple EBOV variants in serum samples, even in individuals undergoing viral clearance and convalescence.

## 4. Discussion

Sensitive and rapid detection methods are crucial for pathogen screening and for restriction of rapid spread of a virus through a naïve population. The epidemic of EBOV disease (EVD) in West Africa (2013–2016) has indeed accelerated efforts to develop different types of diagnostic methods [22]. Molecular methods, such as RT-qPCR, have rightly become the preferred method to diagnose cases of EVD [23,24,25]. In addition, serological diagnostic systems for detection of EBOV antigen or EBOV-reactive antibodies present in patient serum have also been developed [22]. The standard early diagnostic protocol for antigen detection in patient serum is the sandwich ELISA. In this study, we described a sandwich ELISA to detect EBOV sGP in serum samples. The sandwich ELISA showed high sensitivity and specificity in three different animal models.

First, we detected and quantified EBOV sGP from different EBOV-infected cell supernatants and found that the sandwich ELISA has no binding ability to EBOV-C05 due to a single aa substitution (Figure 2 and Figure 3). The aa difference is only observed in EBOV-C05 but not in other EBOVs including EBOV-C07 (Figure 3), EBOV-Makona Liberia 4156, and EBOV-Makona Mali #29, indicating that the sGP of most EBOV variants could be detected by this sandwich ELISA. However, in order to detect EBOV-C05, future work should focus on generation of polyclonal antibodies using the peptides GEWAFWETKKN and GEWAFRETKKN to enable detection sGP of all known EBOV variants.

Continued development of EBOV countermeasures depends heavily on animal models that recapitulate the disease observed in humans. A variety of animal models of EBOV infection have been used for basic research, characterization of pathogenesis, and development of antivirals and vaccines [26]. The dynamics of virus replication is important for evaluation of studies performed using these animal models. With our sandwich ELISA, we quantified EBOV sGP in the serum of three different infected animal species, and the results confirmed that, similar to RT-qPCR and virus titer, the assay can indeed be applied to multiple animal models. Of particular note, the NHP study revealed that EBOV sGP dynamics correlate with virus replication dynamics (Figure 5 and [15]), indicating that this ELISA has the potential to be utilized as a measurement tool for vaccine and drug efficacy.

Rapid diagnostic tests can leverage the same antibody/antigen capture reagents as an ELISA but in a lateral flow strip format, allowing for faster results with minimal specimen processing. For example, the ReEBOV Antigen Rapid Detection Test to detect EBOV-VP40 was the first antigen-based assay to receive FDA and World Health Organization emergency use authorization during the EVD epidemic in West Africa [27]. Several other EBOV rapid diagnostic tests were generated and some of them were approved during the outbreak [28]. A recent study reported generation of an sGP and GP detection kit based on immunochromatography coupled with silver amplification technology [11]. The limit of detection for this assay was 2.21 × 10^4^ genome copies/mL in plasma samples from an infected NHP. In comparison, our sandwich ELISA detected sGP in samples with corresponding viremia as low as 100 TCID_50_ equivalents/mL or 5.6 × 10^3^ TCID_50_/_mL_ [15], suggesting that our system also shows high sensitivity for EBOV sGP. Furthermore, we could detect sGP in the serum of one NHP that had no viremia on day 6 [15] and only showed low levels of viral RNA in the blood (Figure 5, EBOV-Mali #29). This aspect is giving interesting insight into sGP expression dynamics; however, more studies are needed to determine the halflife time of this protein in a convalescent patient. Thus, based on this observation this ELISA could be developed into a diagnostic tool for detection of acute and early convalescent patients.

Our study described here provides a promising start for development of a novel rapid diagnostic test. The capture antibody used in this study (ZGP42/3.7) showed cross-binding ability to all human-pathogenic ebolavirus species [21,29], allowing for development of a detection method for sGP of other ebolaviruses by only changing the detection antibody.
